# Global Seroprevalence (HSV‐2 IgG), Risk Factors, and Outcomes of Genital Herpes Infection During Pregnancy: A Systematic Review and Meta‐Analysis

**DOI:** 10.1155/jp/7595836

**Published:** 2026-04-25

**Authors:** Nader Salari, Ashkan Fatemi Gohar, Fateme Lotfi, Farnaz Jalili, Negar Heidari, Paria Heidari, Masoud Mohammadi

**Affiliations:** ^1^ Department of Biostatistics, School of Health, Kermanshah University of Medical Sciences, Kermanshah, Iran, kums.ac.ir; ^2^ Student Research Committee, Kermanshah University of Medical Sciences, Kermanshah, Iran, kums.ac.ir; ^3^ Department of Medicine, School of Medicine, University of Adelaide, Adelaide, Australia, adelaide.edu.au; ^4^ Department of Health Education and Promotion, School of Health, Kermanshah University of Medical Sciences, Kermanshah, Iran, kums.ac.ir; ^5^ Research Center for Social Determinants of Health, Jahrom University of Medical Sciences, Jahrom, Iran, jums.ac.ir

**Keywords:** Herpes Simplex Virus Type 2, HSV-2 IgG, pregnancy seroprevalence

## Abstract

**Background:**

Genital herpes (Herpes Simplex Virus Type 2) infection (HSV‐2 IgG) is a common infection in adults. Given that the virus can indirectly affect fetal development, the infection is particularly important in pregnant women. The disease can remain latent for extended periods and, in addition to causing chronic and recurrent complications, can sometimes result in miscarriage. This study is aimed at investigating the global seroprevalence, risk factors, and outcomes of HSV‐2 IgG during pregnancy through a systematic review and meta‐analysis.

**Method:**

In this study, the electronic databases, including PubMed, Scopus, Web of Science, Embase, ScienceDirect, and Google Scholar, were systematically searched to find studies reporting the seroprevalence of HSV‐2 IgG until February 2025. A random‐effects model was used for analysis. Heterogeneity between studies was assessed using the *I* ^2^ index. Data analysis was performed using the software Comprehensive Meta‐Analysis (Version 2).

**Results:**

In the analysis of 15 studies with a sample size of 19,197 pregnant women, the global seroprevalence of HSV‐2 IgG in pregnant women was reported as 23.4%. In examining the factors influencing the heterogeneity of the studies and the impact of sample size on this heterogeneity, it was reported that as sample size increased, the seroprevalence of HSV‐2 IgG in pregnant women decreased (*p* < 0.05). Additionally, the findings suggested that with the increase in the year of study, the seroprevalence decreased (*p* < 0.05). In this study, a history of sexually transmitted diseases and having more than one sexual partner were also identified as factors associated with HSV‐2 IgG in pregnancy.

**Conclusion:**

Given the seroprevalence (HSV‐2 IgG), associated factors, and significant complications of contracting this infection during pregnancy, it is recommended that relevant authorities provide the necessary resources to increase awareness among the public and apply appropriate prevention and follow‐up measures to minimize the risk of infection.

## 1. Background

Throughout history, populations have faced various diseases, one of which is HSV‐2 IgG infection [1]. The HSV‐2, similar to other species of herpes simplex that cause oral herpes, belongs to a large family of DNA viruses called the Herpesviridae family [1]. Although the name genital herpes confines the disease caused by the HSV‐2 IgG strain to the genital area, both strains are capable of causing infections in both the genital and oral regions [2]. For example, the HSV‐1 strain can also infect the genital area through oral–genital contact, leading to genital herpes infection; however, genital infection by HSV‐1 and oral infection by HSV‐2 IgG are uncommon [3].

Among the mentioned strains, HSV‐2 IgG has raised increasing concerns [4]. These concerns are particularly significant because if the mother is infected during pregnancy, the infection can be vertically transmitted to the fetus [4].

Diagnosing the symptoms of HSV‐2 IgG infection could be challenging. The difficulty in this diagnosis can be attributed to the asymptomatic nature of HSV‐2 IgG [4]. However, several symptoms can be observed, including inflamed blisters accompanied by the appearance of sores in the genital and anal areas, purulent rashes, ulcers, and, in more advanced cases, the erosion of parts of the genital area such as the vagina, cervix, small and large labia [5, 6]. Additionally, some patients complain of itching and pain in the genital area [5]. The progression of the virus does not stop with these symptoms, as, in some cases, HSV‐2 IgG can create a predisposition to meningitis, encephalitis, or infection of the fetus by the mother [7].

Statistics show that more than 490 million middle‐aged individuals worldwide are infected with this disease, reflecting the widespread nature of HSV‐2 IgG infection [8]. The infection rate of HSV‐2 IgG is estimated to be 31.4% in some countries, such as Haiti [9]. Although the incidence of this infection has been declining over the past two decades [10].

In 2020 alone, 25.6 million individuals aged 15–49 years worldwide were reported to be infected with HSV‐2 IgG [11], and according to estimates by the World Health Organization (WHO), 20 million new cases are added annually to the global population of infected individuals [12].

The complications and risks that genital herpes poses to both the mother and fetus have led us to conduct a review of the existing studies in this field to explore the risk factors and outcomes of this infection. Therefore, the aim of this study is to conduct a systematic review with meta‐analysis to investigate the seroprevalence of HSV‐2 IgG infection during pregnancy, with the information potentially helping the adoption of appropriate health development policies.

## 2. Method

In this systematic review and meta‐analysis, based on the PRISMA statement guidelines, the available evidence regarding the global seroprevalence, associated risk factors, and outcomes of HSV‐2 IgG in pregnant women was reviewed. Our initial search was conducted until February 2025. The search used keywords such as “seroprevalence,” “HSV‐2 IgG,” “herpes simplex virus type 2,” and “pregnancy,” combined with the Boolean operators (AND) and (OR). To find relevant studies, searches were carried out in Google Scholar, PubMed, Web of Science (WoS), Scopus, Embase, and ScienceDirect. It is worth noting that in order to find related articles, reference lists and citations in each article were reviewed, and references from previous studies were also used to identify relevant findings. After identifying the studies, the data were transferred to EndNote, and those studies that reported the global seroprevalence of genital herpes during pregnancy according to the inclusion criteria were declared as accepted studies (Table 1).

**Table 1:** Keywords and search patterns in each database.

Database	Search type	Search strategy
PubMed	Advanced search	• (“seroprevalence”[tiab] OR “outbreak”[tiab] OR “epidemiology”[tiab] OR “Incidence”[tiab] OR “consequence”[tiab] OR “outcome”[tiab] OR “Risk factors”[tiab] OR “related factors”[tiab]) AND (“Herpes Simplex Virus 2”[tiab] OR “Herpes Simplex Virus Type 2”[tiab] OR “HSV‐2 IgG”[tiab] OR “(Herpesvirus 2 (alpha) AND Human)”[tiab] OR “Human Herpesvirus 2”[tiab]) AND (“pregnancy”[tiab] OR “gestation”[tiab])
ScienceDirect	Advanced search	Title, abstract, keywords: (“seroprevalence” OR “outbreak” OR “epidemiology” OR “Incidence” OR “consequence” OR “outcome” OR “related factors” OR “Risk factors”) AND (“Herpes Simplex Virus 2” OR “Herpes Simplex Virus Type 2” OR “HSV‐2 IgG” OR “(Herpesvirus 2 (alpha) AND Human)” OR “Human Herpesvirus 2”) AND (“pregnancy” OR “gestation”)
Scopus	Advanced search	TITLE‐ABS‐KEY (“seroprevalence” OR “outbreak” OR “epidemiology” OR “Incidence” OR “related factors” OR “Risk factors”) AND (“Herpes Simplex Virus 2” OR “Herpes Simplex Virus Type 2” OR “HSV‐2 IgG” OR “(Herpesvirus 2 (alpha) AND Human)” OR “Human Herpesvirus 2”) AND (“pregnancy” OR “gestation”) AND (LIMIT‐TO (LANGUAGE, “English”)
WOS	Advanced search	TS = (“seroprevalence” OR “outbreak” OR “epidemiology” OR “consequence” OR “outcome” OR “related factors” OR “Risk factors”) AND TS = (“Herpes Simplex Virus 2” OR “Herpes Simplex Virus Type 2” OR “HSV‐2 IgG” OR “(Herpesvirus 2 (alpha) AND Human)” OR “Human Herpesvirus 2”) AND TS = (“pregnancy” OR “gestation”)
Embase	Advanced search	(“seroprevalence”:ti,ab,kw OR “outbreak”:ti,ab,kw OR “epidemiology”:ti,ab,kw OR “incidence”:ti,ab,kw OR “consequence”:ti,ab,kw OR “outcome”:ti,ab,kw OR “related factors”:ab,ti OR “Risk factors”:ab,ti) AND (“Herpes Simplex Virus 2”:ti,ab,kw OR “Herpes Simplex Virus Type 2”:ti,ab,kw OR “(Herpesvirus 2 (alpha) AND Human)”:ti,ab,kw OR “HSV‐2 IgG”:ti,ab,kw) AND (“pregnancy”:ti,ab,kw OR gestation:ti,ab,kw)
Google Scholar	Simple search	(“seroprevalence” OR “outbreak” OR “epidemiology” OR “consequence” OR “outcome” OR “related factors” OR “Risk factors”) AND (“Herpes Simplex Virus 2” OR “Herpes Simplex Virus Type 2” OR “HSV‐2 IgG” OR “(Herpesvirus 2 (alpha) AND Human)” OR “Human Herpesvirus 2”) AND (“pregnancy” OR “gestation”)

### 2.1. Inclusion and Exclusion Criteria

#### 2.1.1. Inclusion Criteria for Studies

Inclusion criteria were as follows:

1. Observational studies (cohort, case–control, and cross‐sectional studies) that reported the seroprevalence, associated factors, and outcomes of genital herpes during pregnancy.
2. Studies where the full text was available.
3. Studies published in English.
4. Articles that provided sufficient data (e.g., sample size of individuals with genital herpes during pregnancy).

#### 2.1.2. Exclusion Criteria for Studies

Exclusion criteria were as follows:

1. Case studies.
2. Intervention studies.
3. Studies where the full text was not available.
4. Studies that were not in English.
5. Duplicate studies.
6. Studies that did not provide sufficient data to calculate the overall seroprevalence of genital herpes during pregnancy.

### 2.2. Study Selection

The following steps were used to select relevant studies. Firstly, duplicated studies that were identified in databases were removed. Then, articles were reviewed according to their titles and abstracts, and a number of articles that did not meet the inclusion and exclusion criteria were excluded. Next, the full texts of the remaining studies were reviewed by the researchers, and those that were unrelated to our study based on the inclusion and exclusion criteria were also removed. It is important to note that all stages of reviewing the resource and data extraction were carried out by two researchers independently to avoid bias. In cases where there were discrepancies between the two researchers′ opinions, a third person was consulted to assist with the review of the studies.

### 2.3. Quality Assessment

The Newcastle–Ottawa Scale (NOS) is a quality assessment tool for observational studies that are recommended by the Cochrane Collaboration. The NOS awards up to nine points across three domains: (1) selection of study groups (up to four points), (2) comparability of groups (up to two points), and (3) ascertainment of exposure and outcomes for case–control and cohort studies (up to three points). Studies were classified as high quality (NOS score ≥ 5) or low quality (NOS score < 5). In this meta‐analysis, only studies with an NOS score ≥ 5 were included. [13].

### 2.4. Data Extraction

Two researchers independently extracted the data and entered the required information into a checklist. The checklist included multiple specifications, such as the name of the first author, the year of publication, the study location, sample size, age group or mean age of participants, seroprevalence percentage and number, and the tools used for measuring HSV‐2 IgG.

### 2.5. Statistical Analysis

In this study, data were analyzed using the software Comprehensive Meta‐Analysis (Version 2). The *I* ^2^ test was used to assess heterogeneity between studies, and publication bias was assessed using the Egger test and the Begg and Mazumdar rank correlation test at a significance level of 0.05, as well as a funnel plot.

## 3. Results

**Figure 1:**
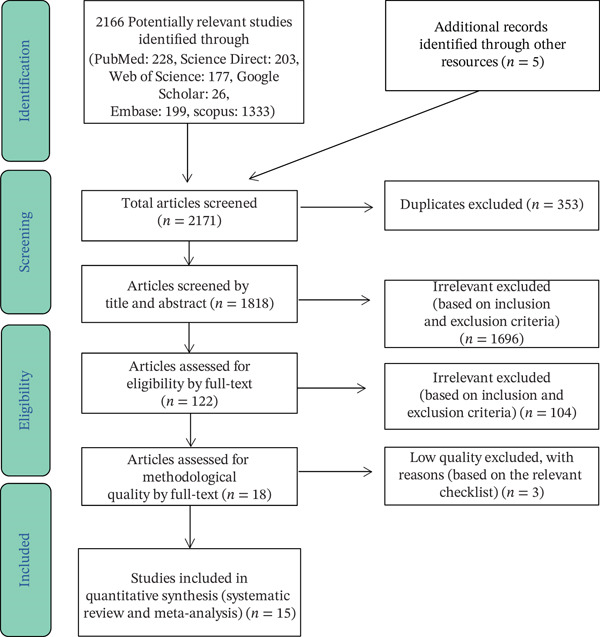
PRISMA flow diagram for study selection.

A total of 2171 records were identified, including 2166 from database searching and five from manual searching, and were imported into EndNote. In the next step, out of 2171 studies, 353 were excluded due to being duplicates, and 1696 studies were excluded based on the inclusion and exclusion criteria after reviewing their titles and abstracts. In the secondary evaluation, 104 studies were excluded after reviewing the full text, based on the inclusion and exclusion criteria. A total of 18 studies remained, three of which were excluded due to low quality. Finally, 15 studies met the inclusion and exclusion criteria and remained in the study. The information from these studies is shown in Table 1. As shown in Table 1, the highest seroprevalence of HSV‐2 IgG was found in a study by Duran in Turkey, which reported a 63.1% seroprevalence of HSV‐2 IgG in pregnancy among 130 samples [14]. The lowest seroprevalence, 6.8%, was reported in a study by Obeid conducted in Saudi Arabia [15]. The majority of accepted studies were from Asia (five studies), while the fewest studies were from America (two studies) (Figure 1 and Table 2).

**Table 2:** Summary of characteristics of included studies of seroprevalence of HSV‐2 IgG in pregnancy.

Author	Year	Country	Study design	Sample size	Age	% Seroprevalence	Testing method	Quality assessment
Dan et al. [16]	2003	Israel	Cross‐sectional	512	16–44	13.3%	ELISA	Moderate
Diamreyan et al. [9]	2021	Nigeria	Cross‐sectional	168	20–49	58.9%	Laboratory analysis for HSV‐2 (IgG)	High
Duran [14]	2003	Turkey	Cross‐sectional	130	17–44	63.1%	ELISA	Moderate
Li et al. [17]	2011	China	Cross‐sectional	1740	21–39	23.56%	ELISA	Moderate
Kucera et al. [18]	2011	Switzerland	Cross‐sectional	1030	14–46	21.2%	ELISA	Moderate
Kurewa et al. [19]	2010	Zimbabwe	Cross‐sectional	691	< 20 & 20–24 & 25–29 & > 30	51.1%	ELISA	Moderate
Biswas et al. [20]	2011	India	Cross‐sectional	1640	≤ 18	8.7%	ELISA	Moderate
LeGoff et al. [21]	2007	France	Cross‐sectional	76	20–46	26.3%	Genital+blood samples	High
Domercant et al. [22]	2017	Haiti	Cross sectional	1000	15–49	31.4%	ELISA	Moderate
Yahya‐Malima et al. [23]	2008	Tanzania	Cross‐sectional	1296	15–49	20.7%	ELISA	Moderate
Xu et al. [24]	2007	United States	Cross‐sectional	626	12–59	22%	Immunodot assays	High
Patton et al. [25]	2018	United States	Cross‐sectional	8124	20–39	21.1%	Serologic testing	Moderate
Obeid [15]	2007	Saudi Arabia	Cross‐sectional	459	17–49	6.8%	ELISA	High
Radoi et al.^a^ [1]	2024	Romania	Cross‐sectional	355	15–44	16.16	ECLIA	High
Radoi et al.^b^ [1]	2024	Romania	Cross‐sectional	1350	14–47	12.43%	Immune chemiluminescence	High

Abbreviations: ECLIA, electrochemiluminescence immunoassay; ELISA, enzyme‐linked immunosorbent assay; IgG, immunoglobulin G.

^a^Enzyme‐linked immunosorbent assay.

^b^Immunoglobulin G.

**Figure 2:**
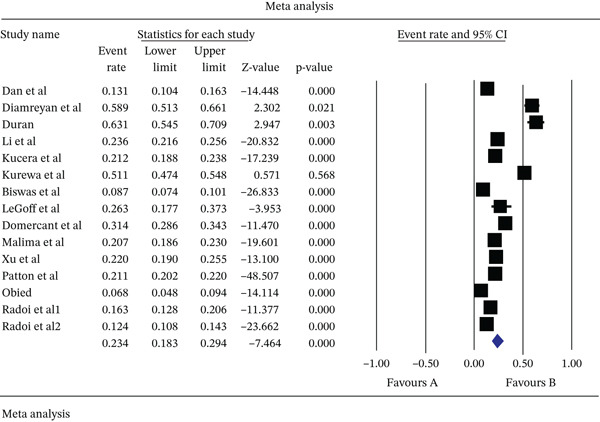
Funnel plot for publication bias assessment in the reviewed studies.

**Figure 3:**
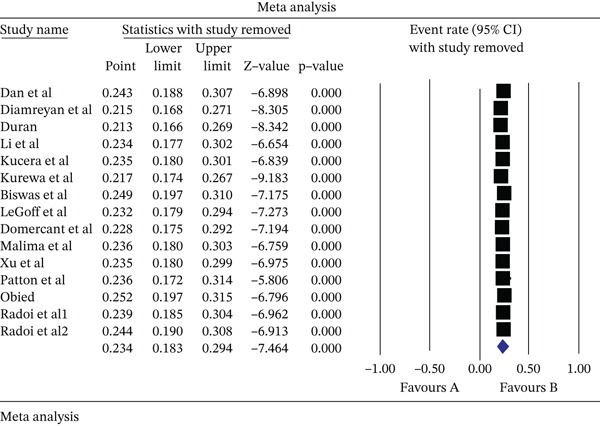
Results of sensitivity analysis in studies analyzed in meta‐analysis.

**Figure 4:**
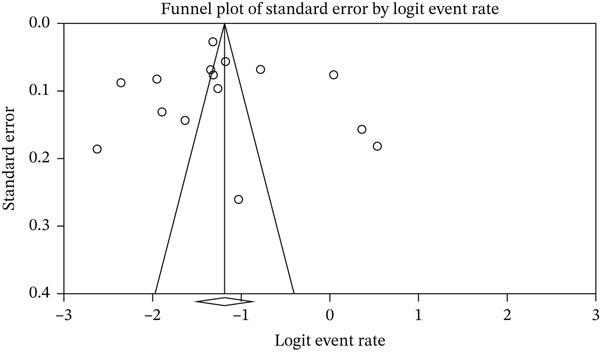
Forest plot of HSV‐2 IgG seroprevalence in pregnant women based on the random‐effects model.

In the review of 15 studies with a sample size of 19197 pregnant women, the *I* ^2^ heterogeneity test showed high heterogeneity (*I* ^2^: 98.3%; *τ* ^2^: 0.364), and based on that, a random‐effects model was used to analyze the results. According to the meta‐analysis, the seroprevalence of HSV‐2 IgG in pregnant women was reported as 23.4% (95% CI: 18.3–29.4) (Figure 2). The results of the sensitivity analysis are reported in Figure 3. Moreover, the publication bias assessment through the Egger test showed no evidence of publication bias in the studies (*p*: 0.772). The results of the Begg and Mazumdar rank correlation test also showed no significant publication bias (0.960) (Figure 4). However, with the presence of 15 studies and very high heterogeneity, the power of the tests to examine publication bias is limited.

**Figure 5:**
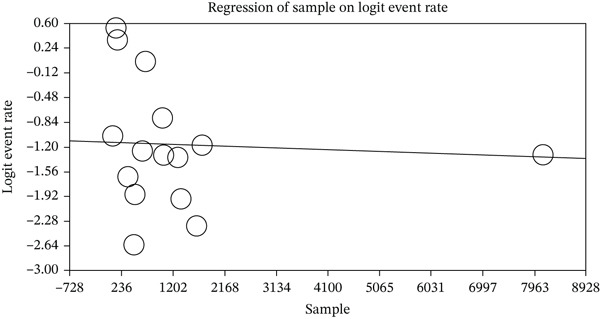
Metaregression of the impact of sample size on the seroprevalence of HSV‐2 IgG in pregnant women.

**Figure 6:**
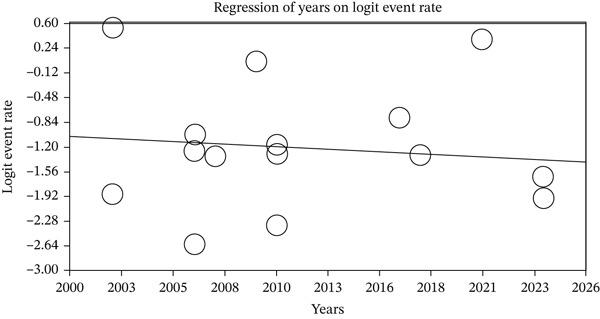
Metaregression of the impact of year of study cohort on the seroprevalence of HSV‐2 IgG in pregnant women.

After examining the factors influencing the heterogeneity of studies and the impact of sample size on this heterogeneity, it was reported that as sample size increased, the seroprevalence of HSV‐2 IgG in pregnant women decreased (*p* < 0.05) (Figure 5 and Table 3). Meanwhile, as the year of study increased, the seroprevalence of HSV‐2 IgG in pregnant women also decreased (*p* < 0.05) (Figure 6 and Table 3).

**Table 3:** Reporting the results of metaregression analysis based on sample size and year of study.

Variable	Point estimate	Standard error	Lower limit	Upper limit	*Z*‐value	*p*value
Sample	−0.00002	0.00001	−0.00003	−0.00001	−4.704	0.000
	−1.14	0.028	−1.2022	−1.0908	−40.34	0.000
Years	−0.014	0.0036	−0.021	−0.0075	−4.05	0.00005
	28.11	7.2503	13.908	42.329	3.878	0.00011

### 3.1. Factors Affecting HSV‐2 IgG

Based on relevant studies, several factors may be associated with HSV‐2 IgG infection and can be considered risk factors for contracting this infection. A brief explanation of these factors is provided, with their characteristics organized in Table 4.

**Table 4:** Characteristics of studies included in the systematic review.

Author	Year	Region	Type of study	Sample size	Age	*p*value	OR (95% CI)	Risk factor	Testing method
Dan et al. [16]	2003	Israel	Cross‐sectional	512	14–44	< 0.0001	3.32 (10.442)	Lifetime sex partner [2–10]	ELISA
						< 0.0005	5.06	STD diagnosis history	
Biswas et al. [20]	2011	India	Cross‐sectional	1640	≥ 18	0.04	2.5 (1.1–6.1)	Multiple sexual partners	ELISA
						< 0.001	4.7 (2.5–8.9)	Lack of condom use	
						0.04	9.6 (1.1–81.6)	First sexual intercourse < 18 years (coitarche age)	
						0.02	2.2 (1.3–10.7)	Low education level	
						0.001	2.2 (1.4–3.3)	Low income	
						0.01	3.4 (1.3–8.8)	Regular employment income	
						0.02	1.7 (1.1–2.6)	Family structure/type	
Munjoma et al. [26]	2010	Zimbabwe	Cohort	340	20–28	< 0.001	1.7 (1.4–2.1)	Trichomonas vaginalis infection	ELISA
						0.025	1.8 (1.4–2.4)	History of multiple sexual partners in the previous 12 months	
Vilibic‐Cavlek [3]	2024	Croatia	Cross‐sectional	667	16–47	< 0.001	1.19 (1.09–1.30)	Age	Immunoblot assay & ELISA

### 3.2. Sexual Partners

According to studies, having more than one sexual partner plays a significant role in transmitting the HSV infection (Table 4) [16, 20, 26].

### 3.3. Sexually Transmitted Diseases (STDs)

A history of sexually transmitted infections can also be an important factor in the susceptibility to HSV‐2 IgG (Table 4) [16, 26].

### 3.4. Other Factors

It is also worth noting that factors such as low education levels; poor economic status; lack of use of barrier contraception, such as condoms; and others can significantly contribute to the likelihood of contracting herpes (Table 4) [20].

## 4. Discussion

The aim of this study was to determine the global seroprevalence of HSV‐2 IgG during pregnancy, and based on the analyses conducted, the seroprevalence of this disease during pregnancy was found to be 23.4%. The analysis revealed that, generally, as the sample size increased, the seroprevalence of HSV‐2 IgG in pregnant women decreased. It is also worth noting that over time, with the increase in the year of study, the seroprevalence of HSV‐2 IgG during pregnancy has also decreased.

Estimates suggest that the seroprevalence of HSV‐2 IgG in middle‐class primary care settings is 25%, with the severity of the disease depending on the individual′s immunity [27, 28]. HSV‐2 IgG penetrates through mucous membranes or damaged skin, eventually reaching the nerve cells to initiate a latent infection [29]. The term “non‐primary episode” occurs when an individual with antibodies to one type of HSV encounters a different type; in other words, a person with antibodies against HSV‐1 becomes infected with HSV‐2, or the reverse may occur. “Recurrent infection” is another term used for when antibodies already exist against the same type of HSV [30].

Antiviral treatments such as acyclovir, valacyclovir, and penciclovir can be used in the treatment of HSV infection [31]. Currently, no vaccines have been developed to immunize individuals against this infection, largely because of the virus′s latency and its ability to evade immunity [32]. In Iran, herbal treatments named Melissa and Myrtoplex are used against HSV‐2 infection [31].

We calculated the seroprevalence of HSV‐2 in pregnant women to be 23.4%. Our findings differed from some studies that reported varying seroprevalence rates for HSV infection. However, despite these differences, some studies have supported our seroprevalence rates by having results similar to ours. For example, in a study by Li et al. conducted in China, the seroprevalence of HSV‐2 during pregnancy was reported as 23.56% [17]. Another study by Kucera et al. on 1030 samples found a seroprevalence of 21.2% for HSV‐2 infection during pregnancy [18]. LeGoff et al. also estimated the seroprevalence to be 26.3% in a sample of 76 individuals [21], and the seroprevalence rates in the studies by Xu et al. in the United States and Yahya‐Malima et al. in Tanzania were 22% and 20.7%, respectively [23, 24].

In contrast to the studies reporting low seroprevalence, some studies have reported higher seroprevalence rates. For instance, a study done by Duran reported a seroprevalence of 63.1% in pregnant women aged 14–44 years [14]. This seroprevalence rate is similar to the 58.9% seroprevalence found in the study by Diamreyan et al., which focused on pregnant women aged 20–49 years [9], both highlighting a significant seroprevalence. Moreover, a study by Kurewa et al. reported a 51.1% seroprevalence in 691 samples, which is higher than our findings [19].

One of the limitations of this meta‐analysis is that only studies published in English were included in the analysis, which may result in overlooking other reports and findings in other languages. It is recommended that future studies also include research published in other languages. Moreover, the number of studies in this field was limited, posing another challenge during the conduction of this study. Due to the high heterogeneity, we used metaregression tests, but not all studies reported clear results regarding other heterogeneity‐causing variables, so we were unable to use subgroup analysis. Therefore, the study results need to be interpreted with extreme caution.

## 5. Conclusion

Given the 23.4% seroprevalence of HSV‐2 IgG infection in pregnant women, it is important that policymakers and healthcare authorities consider the results of this meta‐analysis in an effort to increase health literacy and raise awareness within the community. This will pave the way for creating the necessary conditions to reduce the seroprevalence rate of the disease. Consequently, the reduced number of HSV infections will reduce the complications associated with this infection.

**Nomenclature**
PRISMA: Preferred Reporting Items for Systematic Reviews and Meta‐Analyses

## Author Contributions

N.S., A.F.G., M.M., and F.L. contributed to the design, while M.M. performed statistical analysis and participated in most of the study steps. M.M., P.H., and N.H. prepared the manuscript. M.M., F.J., P.H., and N.H. assisted in designing and interpreting the study.

## Funding

This study is supported by the Deputy for Research and Technology, Kermanshah University of Medical Sciences (IR) (4040410).

## Disclosure

Deputy for Research and Technology, Kermanshah University of Medical Sciences, has no role in the study process.

## Ethics Statement

Ethics approval was received from the ethics committee of deputy of research and technology, Kermanshah University of Medical Sciences (IR.KUMS.REC.1404.209).

## Consent

The authors have nothing to report.

## Conflicts of Interest

The authors declare no conflicts of interest.

## Data Availability

Datasets are available through the corresponding author upon reasonable request.
